# Unveiling the invisible: receivers use object weight cues for grip force planning in handover actions

**DOI:** 10.1007/s00221-024-06813-y

**Published:** 2024-03-18

**Authors:** L. Kopnarski, J. Rudisch, D. F. Kutz, C. Voelcker-Rehage

**Affiliations:** Department of Neuromotor Behavior and Exercise Institute of Sport and Exercise Sciences, University of Münster, Münster, Germany

**Keywords:** Object handover, Joint action, Grasp, Lift, Weight anticipation, Grip force

## Abstract

Handover actions are part of our daily lives. Whether it is the milk carton at the breakfast table or tickets at the box office, we usually perform these joint actions without much conscious attention. The individual actions involved in handovers, that have already been studied intensively at the level of individual actions, are grasping, lifting, and transporting objects. Depending on the object’s properties, actors must plan their execution in order to ensure smooth and efficient object transfer. Therefore, anticipatory grip force scaling is crucial. Grip forces are planned in anticipation using weight estimates based on experience or visual cues. This study aimed to investigate whether receivers are able to correctly estimate object weight by observing the giver’s kinematics. For this purpose, handover actions were performed with 20 dyads, manipulating the participant role (giver/receiver) and varying the size and weight of the object. Due to the random presentation of the object weight and the absence of visual cues, the participants were unaware of the object weight from trial to trial. Kinematics were recorded with a motion tracking system and grip forces were recorded with customized test objects. Peak grip force rates were used as a measure of anticipated object weight. Results showed that receiver kinematics are significantly affected by object weight. The peak grip force rates showed that receivers anticipate object weight, but givers not. This supports the hypothesis that receivers obtain information about the object weight by observing giver’s kinematics and integrating this information into their own action execution.

## Introduction

The term *handover* describes a joint action between two actors in which an object is transferred from one person to another (Kopnarski et al. 2023b). Handover actions are part of people’s everyday life and are usually performed without requiring much conscious attention. Yet unconsciously, each actor must plan and execute different components of the joint action, such as reaching, grasping, lifting, and transporting the object. In addition, joint action coordination is facilitated through anticipation. For example, the handover position and time must be anticipated, most likely using the giver’s velocity profile during object transport (Mason and MacKenzie 2005). Likewise, the anticipation of object properties (such as its weight) might be integrated into one’s own action plan for grasping the object (Kopnarski et al. 2023a). In other words, the receiver may use the information (implicitly) obtained by observing the giver’s kinematics to adjust their own grip force scaling.

“Joint action can be regarded as any form of social interaction whereby two or more individuals coordinate their actions in space and time to bring about a change in the environment” (Sebanz et al. 2006, p. 70). The success of joint actions depends on, among other things, (i) shared representations, (ii) anticipation of co-actor behavior, and (iii) continuous integration of the anticipated and the monitored information (Sebanz et al. 2006; Sebanz and Knoblich 2021). The term ‘shared representations’ is used to describe when, in a joint action, an actor not only plans their own action execution but creates a mental representation of the co-actor’s action as if they were executing the actions of the other (Kourtis et al. 2014; Schmitz et al. 2017, 2018; Sebanz et al. 2003; Sebanz and Knoblich 2021; Vesper et al. 2013). Regarding the shared representation in a handover action, the receiver forms (i) a mental representation of the giver’s actions (reaching to the object, grasping and moving it toward the receiver, and releasing grip force) (Becchio et al. 2008; Cini et al. 2019; Gonzalez et al. 2011; Meyer et al. 2013). Based on this representation, (ii) anticipations are made regarding the giver’s behavior (Cini et al. 2019; Controzzi et al. 2018; Gonzalez et al. 2011; Huber et al. 2013; Mason and Mackenzie 2005). By monitoring the movement of the giver, (iii) a continuous comparison is made between the anticipated and the observed behavior (Cini et al. 2019; Controzzi et al. 2018; Huber et al. 2013). If there are discrepancies between the anticipated and observed behavior, the receiver’s action plan needs to be adjusted. This comparison between anticipation and observation may provide information about object properties that are not available until object lift-off (e.g., weight). For example, if the receiver anticipates the giver’s motor execution as the giver grasps and lifts the object, a deviation from this anticipation (e.g., an unexpectedly fast object lift) may lead to the inference that the object weight is lower than anticipated.

Joint handover actions can be divided into consecutive phases: (1) reach and grasp (begins when the giver reaches for the object), (2) object transport (begins when the object lifts off), (3) object transfer (begins when the receiver makes initial contact with the object), and (4) end of handover (begins when the giver loses contact with the object) (Kopnarski et al. 2023b). The first two phases have already been intensively researched at the individual action level. Thus, it is already known how various object properties (e.g., size, weight) affect the movement of the actors during object grasping and lifting (Flanagan et al. 2006). Movement trajectories that may provide information about the object’s weight are shaped by the grip and load forces produced when objects are grasped and lifted because the required grip and load forces are primarily determined by the object’s properties, in particular its weight (Hermsdörfer 2009; Schneider and Hermsdörfer 2016; Wing 1996). The heavier the object, the greater the grip and load forces that are required to overcome gravity. Dexterous and efficient grasping and lifting of objects is facilitated through assumptions about the object’s weight and the anticipatory control of the grip forces (Flanagan et al. 2009; Flanagan and Wing 1997b; Johansson and Westling 1988). When the object weight is known, the grip and load forces are anticipated so that the grip force can be adapted to the object weight with heavier objects leading to a higher peak grip force rate (Gordon et al. 1991a; Hermsdörfer et al. 2011; Johansson and Westling 1988). Several studies have indicated that not only prior experience (Flanagan et al. 2008, 2009; Gordon et al. 1993), but also visual cues, such as the object’s size, inform weight anticipation (Gordon et al. 1991a, c; Hermsdörfer et al. 2011; Johansson and Westling 1988). If the object is heavier than expected, the grip force rate is scaled erroneously and has to be increased before the object can be lifted. When assessing whether and, if so, to what extent anticipation matches object weight, the peak of the grip force rate was identified as the most reliable parameter (Hermsdörfer et al. 2011). In contrast to the other force parameters examined, it showed no change when the object weight was manipulated without the participant’s knowledge (Gordon et al. 1991a, c; Hermsdörfer et al. 2011; Johansson and Westling 1988; Li et al. 2009; Nowak et al. 2007). When an object is heavier than expected, the load and grip forces have to be adjusted after initial object contact, i.e., the time from initial contact between fingers and object until the object lift-off is extended: This is referred to as lift delay. When an object is lighter than expected, the initially excessive grip force rate is reduced and the excessive load force results in an earlier object lift-off, i.e., a shortened lift delay (Hermsdörfer et al. 2011; Johansson and Westling 1988; Weir et al. 1991).

Another factor that is influenced by a mismatch between expectation and object weight, is the maximum lift velocity, i.e., the greater the object weight, the lower the lift velocity (Johansson and Westling 1988; Kopnarski et al. 2023a). This relationship between object weight and the grip and load forces not only applies in pure lifting tasks but also in joint actions such as in handover tasks. Both, givers and receivers must produce adequate grip and load forces that correspond to the object weight. First, the giver, who grasps and lifts the object in both the reach and grasp phase and the transport phases, needs to overcome the gravitational force acting on the object. As described above, inaccurate load forces can be detected through a prolonged or shortened lift delay. Then, when transporting the object to the handover position (transport phase), inaccurate load forces lead to a lower or higher maximum lift velocity. In the subsequent transfer phase, the receiver must produce accurate grip forces in order to achieve a smooth handover and take control of the object completely (Kopnarski et al. 2023b). This means that when lifting objects of unknown weight, both the lift delay and the maximum lift velocity of the person who lifts the object (in handover actions this is the giver) depend on the object weight. Previous studies have shown that people are able to estimate object weight by observing another actor (Hamilton et al. 2007). In a further study, it was shown in a sequential joint replacement task that the second actor shows lower surprise effects in relation to an object weight change than the first actor (Meulenbroek et al. 2007). This indicates that a second actor has information about the object weight from the observation of the first actor. This ability might be used by receivers in handover actions to adjust their initial grip force scaling to the object weight. Assuming so, this would mean that in addition to one’s previous experience (Flanagan et al. 2008, 2009) and cues including size (Cole 2008; Gordon et al. 1991a, b; Li et al. 2009) and material (Buckingham et al. 2009; Flanagan et al. 1995; Flanagan and Wing 1997a), observing other persons’ actions can also directly influence one’s assumptions about an object’s properties and, therefore, their plan during joint actions.

In summary, the knowledge about object weight might be applied by a receiver in the object transfer phase of a handover action. That means that non-apparent object properties, such as object weight, influence the kinematics of an actor during object manipulation (Hermsdörfer et al. 2011; Johansson and Westling 1988; Kopnarski et al. 2023a; Weir et al. 1991). Further, observers seem to be able to determine information about non-apparent object properties from actor kinematics (Hamilton et al. 2007). What is currently unknown is whether this ability to anticipate object properties by just observing an actor is also employed in the observer’s action planning and whether receivers use this in handover actions. This successful anticipation would be evident in adjustments to actions related to object weight. Thus, this would be measurable in the receiver’s peak grip force rate in the transfer phase of the handover action.

The aim of this study was to investigate whether receivers in handover actions can anticipate the weight of an object by observing the giver’s kinematics and use this information to adapt their own action execution in relation to object weight. Hence, we studied whether the giver’s observable action execution (lift delay, maximum lift velocity) differs systematically as object weight is varied. We hypothesized that (1) the lift delay of the giver increases with increasing object weight, and (2) the maximum lift velocity of the giver decreases with increasing object weight. Further, we examined whether object weight is appropriately estimated by the receiver based on the observation of the giver’s movement. For this reason, we investigated the peak grip force rate of the receiver within the object transfer phase as a function of object weight. We hypothesized that (3) the receiver’s peak grip force rate increases with increasing object weight. As a proof of concept, the peak grip force rate of the giver was in addition investigated. Further we expected that (4) the giver’s peak grip force rate is higher for large objects than for small ones and that weight has no effect on giver’s peak grip force rate.

## Methods

### Participants

Forty participants (31 female, 9 male) aged 22.6 ± 2.5 (18–28) years attended the experiment and gave informed consent for their voluntary participation. Thirty-nine participants were classified as right-handed (score &gt; 40) according to the Edinburgh Handedness Inventory (Oldfield 1971) and one participant was classified as ambidextrous (score = 29.4). All had normal or corrected-to-normal vision and reported no psychiatric or neurological disorders or orthopedic limitations of the upper extremities. Participants received either course points or 10 €/hour for their participation. This study was approved by the Ethics Committee of the Chemnitz University of Technology (Germany), Faculty of Behavioral and Social Sciences, on July 12, 2019 – number V-343-17-CVR-SFB\_A01-24062019.

### Materials

#### Motion tracking

**Fig. 1:**
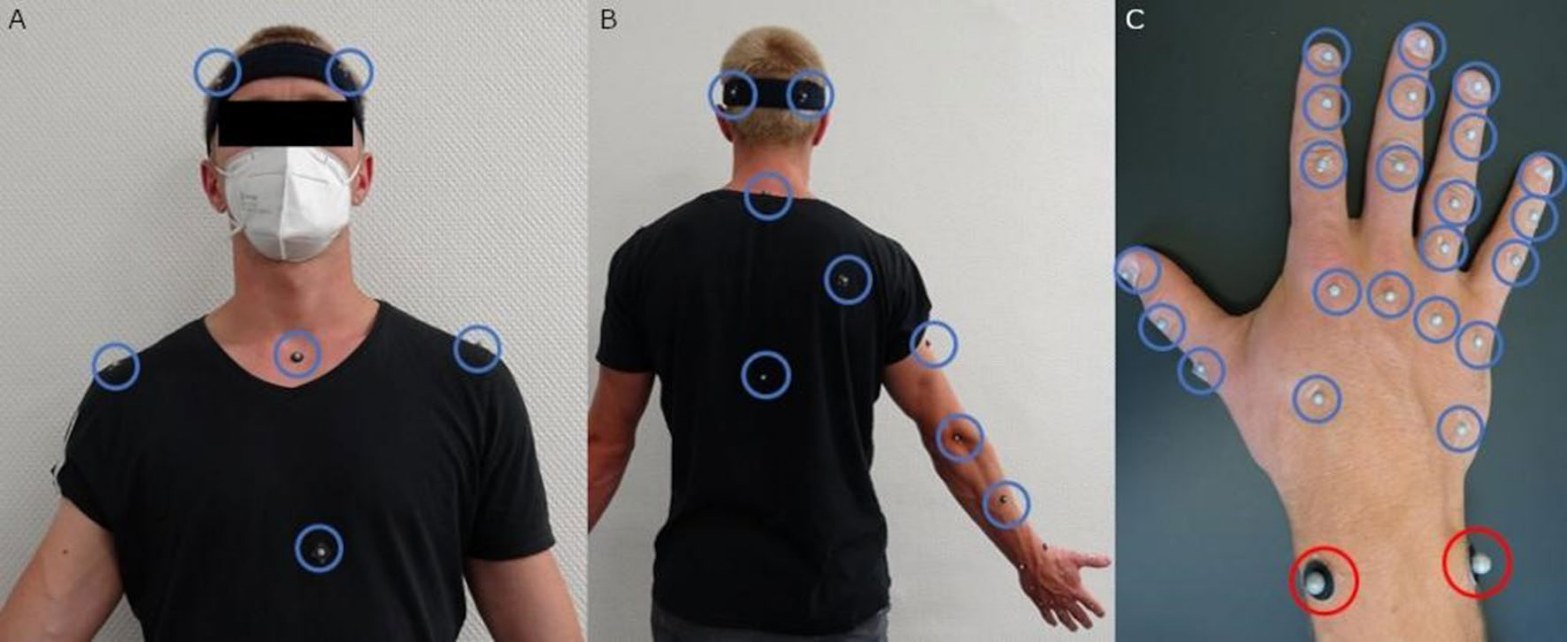
Marker set attached to the upper body and right hand of a participant. The red circles highlight the markers used for this study

A passive marker-based optical motion capture system (Vicon Motion Systems Ltd, Oxford, UK) with 10 cameras was used to record the movement of each participant’s wrist and the object’s kinematics at a sampling frequency of 100 Hz. The objects were tracked by six built-in, active markers (infrared LEDs embedded) detectable in the Vicon system. A total of 38 reflective markers were attached to each participant’s upper body (see Fig. 1). Markers with a diameter of 6 mm were used for the head, trunk, shoulders, and right arm and arranged according to the Plug-In Gait model (Vicon 2021). On the right hand, 22 markers with a diameter of 4 mm were applied according to the Hand model (GPEM). For this study, only the position data of the two markers at the distal end of the radius and ulna were used to calculate the giver’s maximum lift velocity in the object transport phase (Fig. 1C, red circles).

#### Test objects

**Fig. 2:**
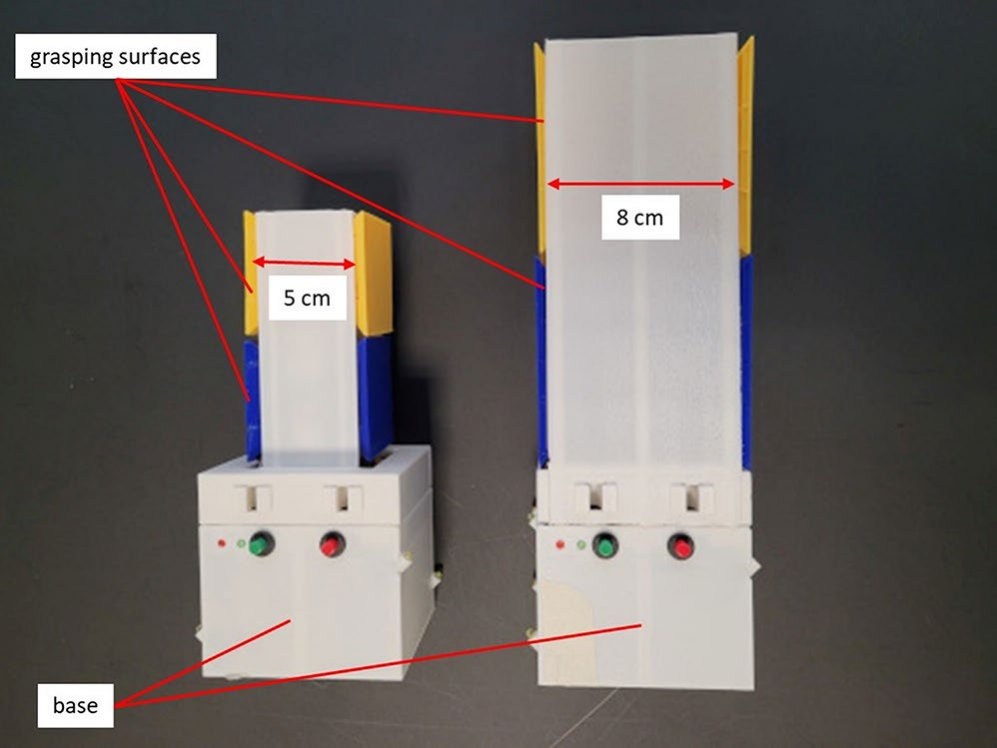
Small (left) and large (right) objects with a base of 8 cm × 8 cm × 8 cm and grasping surfaces of 5 cm × 5 cm × 5 or 8 cm × 8 cm × 8 cm, respectively. The weights were alternately attached in the base, resulting in three different object weights (400 g, 700 g, 1000 g)

The acquisition of the grip force data was conducted using self-constructed 3D-printed test objects. Two different test objects were used (see Fig. 2). The grasping surfaces, which differ in size and distance from each other (5 cm × 5 cm × 5 cm; 8 cm × 8 cm × 8 cm) between the two objects, were located above a body and arranged one above the other (Kutz et al. 2023). Four integrated 3D force-torque sensors (Type 1B-S, Zemic Europe B.V., Etten-Leur, Netherlands) under the grasping surfaces measured the grip force of the giver and receiver separately. Force data was recorded with a sampling rate of 100 Hz. Both objects had an identical body (8 cm × 8 cm × 8 cm), which allowed for the easy and quick attachment of weights inside the object and prevented the participant from seeing which weight was attached. Three different object weights were used, and the overall weights of the whole object did not differ between the small and large objects: light = 400 g, medium = 700 g, and heavy = 1000 g. Six infrared LEDs embedded in the base allowed the object’s motion to be tracked using the Vicon system and the force and kinematic data to be synchronized. A flashing LED light indicated the start of the recording of the force data in the motion tracking recordings.

### Procedure

**Fig. 3:**
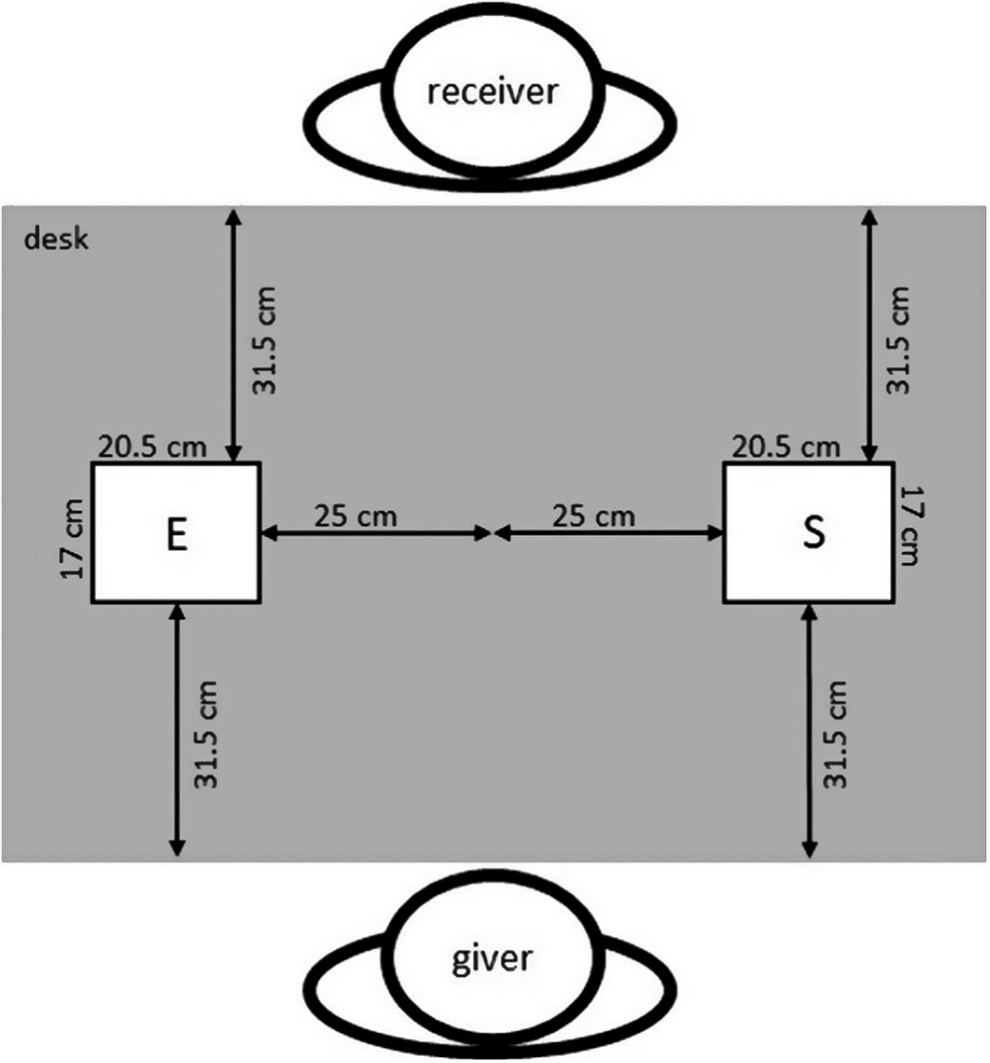
Experimental setup. S indicates the object start position, E indicates the object end position

Two participants were randomly paired and invited to the same session. Reflective markers were attached to both participants according to the model above described. Subsequently, the participants sat opposite each other at a table (see Fig. 3). The height-adjustable stools without a backrest were adapted so that the participant’s elbows were bent about 90° when their forearms were placed on the table (length = 120 cm, width = 80 cm, height = 78 cm) and the palms of their hands rested flat on the table. Participants were asked to take this rest position at the beginning and end of each trial. One test session consisted of four blocks (30 trials each), where one participant was assigned the role of the giver and one the role of the receiver. After half of the trials (after 2 blocks of 30 trials each) the participants switched roles. The condition *object size* was presented in a block design, with the order (whether starting with the small or large object) counterbalanced across all dyads. The condition *object weight* was presented in a pseudo-random order and balanced within a block so that each object weight condition was presented 10 times per block.

Across all trials, each condition was performed 10 times (2 roles x 2 object sizes x 3 object weights) resulting in four blocks and 120 trials. The whole procedure took about 2.5 h.

The changing of weights and object lifting was concealed from the participants by a visual screen. At the beginning of a trial, the object was placed on a foam pad (17 cm x 20.5 cm) and fixed centrally to the table on the right-hand side of the giver (see Fig. 3). The participants were instructed to perform a handover action as naturally as possible. After an acoustic signal, the giver grasped the object at the lower grasping surfaces (see Fig. 2 blue surfaces) and handed it over to the receiver, who grasped it at the upper grasping surfaces (see Fig. 2 yellow surfaces). The receiver then placed the object on a foam pad on the other side of the table (see Fig. 3) ending the trial.

### Data analysis

The preprocessing of the data and statistical analyses were performed using the R 4.2.1 base package (R Core Team 2022).

#### Preprocessing and outcome variables

**Fig. 4:**
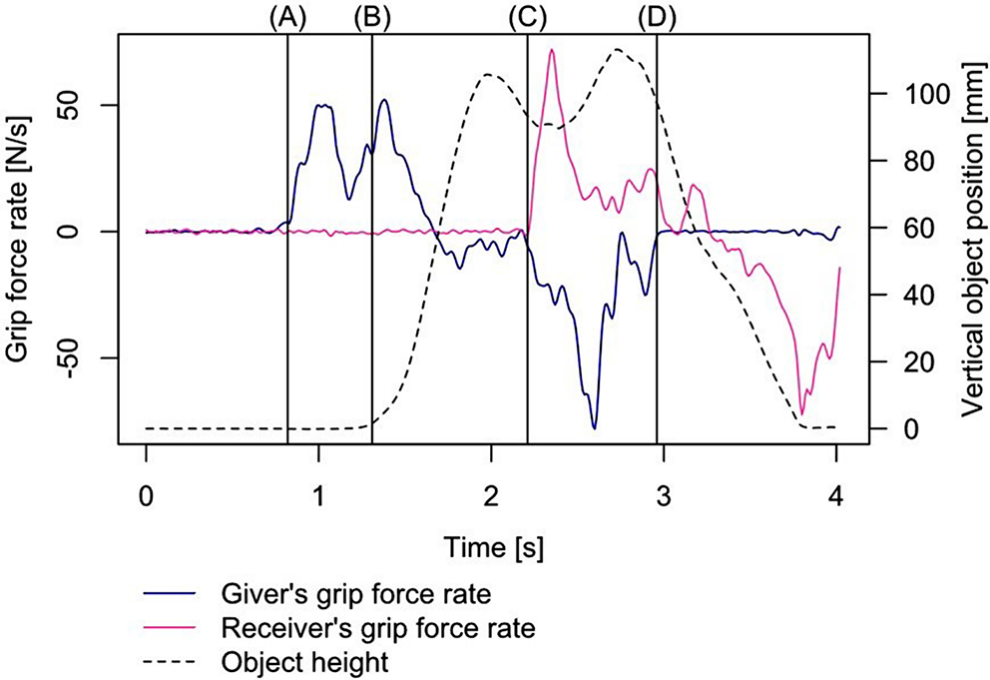
Phase division. The vertical lines indicate the phase dividing events: (**A**) Initial contact between giver and object, (**B**) start of the transport phase, (**C**) end of the transport phase/start of the transfer phase, and (**D**) end of the transfer phase

First, the raw kinematic (mere position coordinates) and force data was filtered using a second order low pass Butterworth filter with a cut-off frequency of 15 Hz. To determine the dependent variables, the data were divided according to the phase model developed in Kopnarski et al. (2023b), which consists of the phases “reach and grasp”, “object transport”, and “object transfer” (see Fig. 4). In order to select appropriate thresholds for the signal-to-noise ratio to separate the phases, manual parameter tuning was used, whereby each individual trial was visually verified for coherence. From the *reach and grasp phase*, only the grasp action is required to determine the lift delay. The start of this is determined by the first contact between the giver and the object, which is defined as the earliest data frame detected with a force change of > = 0.07 N. This phase ends as soon as the object position changes at least 2 mm in the vertical direction. The time between giver-object initial contact and the vertical position change of the object represents the lift delay. This object position change signals the start of the *object transport phase* which then ends with the first contact between the receiver and the object. Since it is possible that an initial collision may occur when the receiver reaches the object but this cannot be defined as grasping, a higher force change threshold of > = 0.09 N was used to determine the end of the *object transport phase*. Within the *object transport phase*, the maximum velocity of the giver’s wrist was determined by deriving the position of the center point between the two markers. To determine the peak grip force rate, the first derivative of the grip forces was used, to which the *findpeaks* function from the *pracma* package (Borchers 2022) was applied. To exclude small initial collisions between participant and object at the beginning of the *grasp/transfer phase*, a threshold of at least 30 N/s was defined. If two or more peaks were identified, only the first peak was used as subsequent peaks were considered corrective actions (Hermsdörfer et al. 2011; Johansson and Westling 1988). When calculating the receiver’s peak grip force rate, there were rare cases, (34 of 2388) for which no peak in the transfer phase could be identified. In these cases, the absolute maximum of the grip force rate within the transfer phase was taken as the receiver’s peak grip force rate.

### Statistical analysis

Given the high individuality of the data, all parameters of interest were z-score normalized on the participant level with the *scale* function. A within-subject factor (size, weight) ANOVA was performed using the R package *ez* (Lawrence 2016). Since the ANOVA with and without consideration of outliers leads to the same result, the outliers were included in the results presented. Post hoc comparisons were carried out using t-test and Cohen’s d for effect sizes.

## Results

The data collection from the 40 participants took place between December 2021 and April 2022. A total of 2400 trials were recorded with 20 participant dyads (120 trials per dyad). However, 12 trials had to be rejected due to technical problems meaning that 2388 trials were included in the analyses. Figure 4 shows exemplary profiles of the grip force rates of the giver and receiver as well as the vertical object displacement of a single trial using the small object and heavy weight.

### Grip forces

**Fig. 5:**
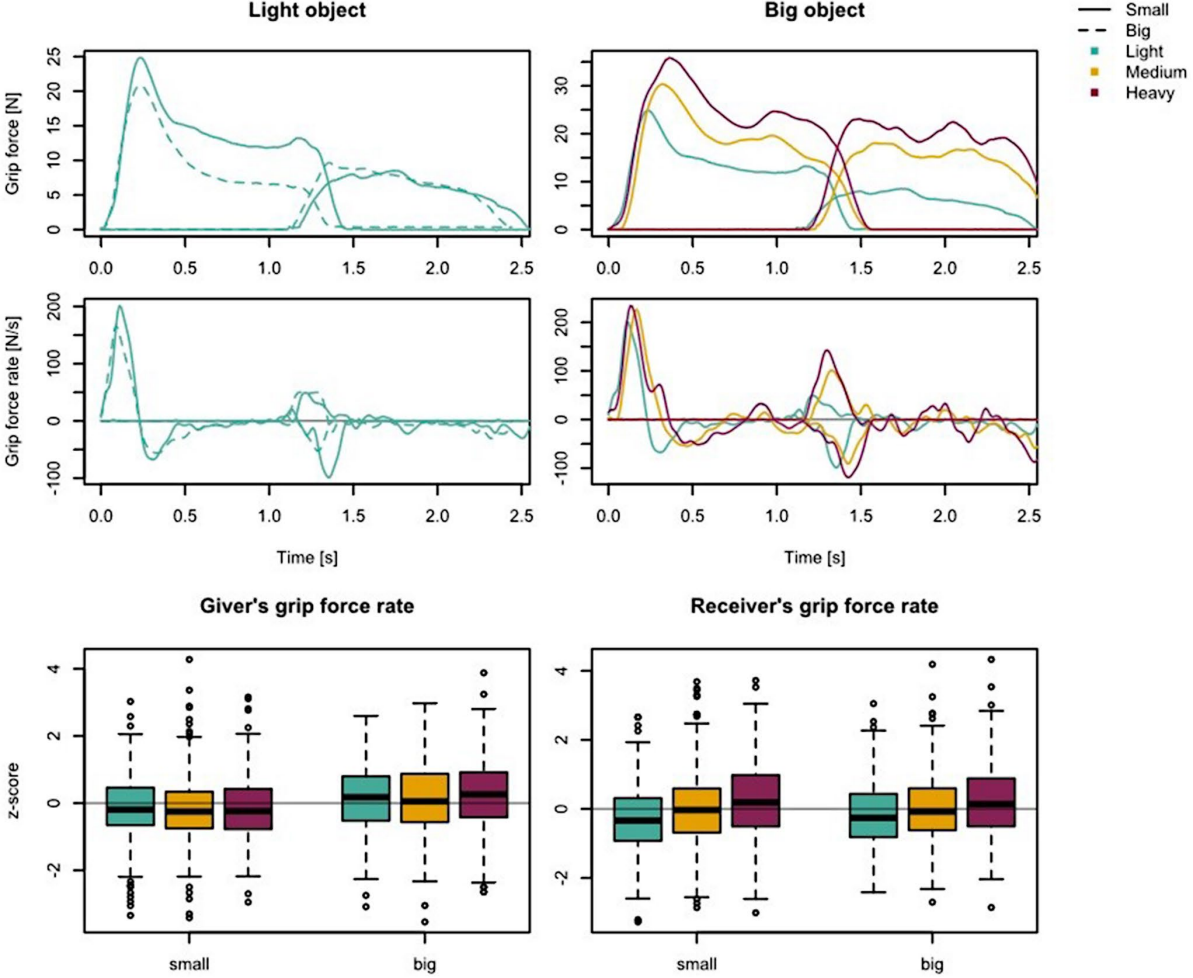
The plotted data are from one dyad (giver and receiver) and aligned to the time of initial contact between giver and object. The left column shows the two trials using the light object weight and the two different object sizes. The right column shows three trials using the big object and the three different object weights (400 g, 700 g, 1000 g). At the bottom the boxplots showing the giver’s and receiver’s z-score normalized grip force rates. Values greater/smaller than 1.5 time of the interquartile range of the third/first quartile were marked by circles as outliers

**Fig. 6:**
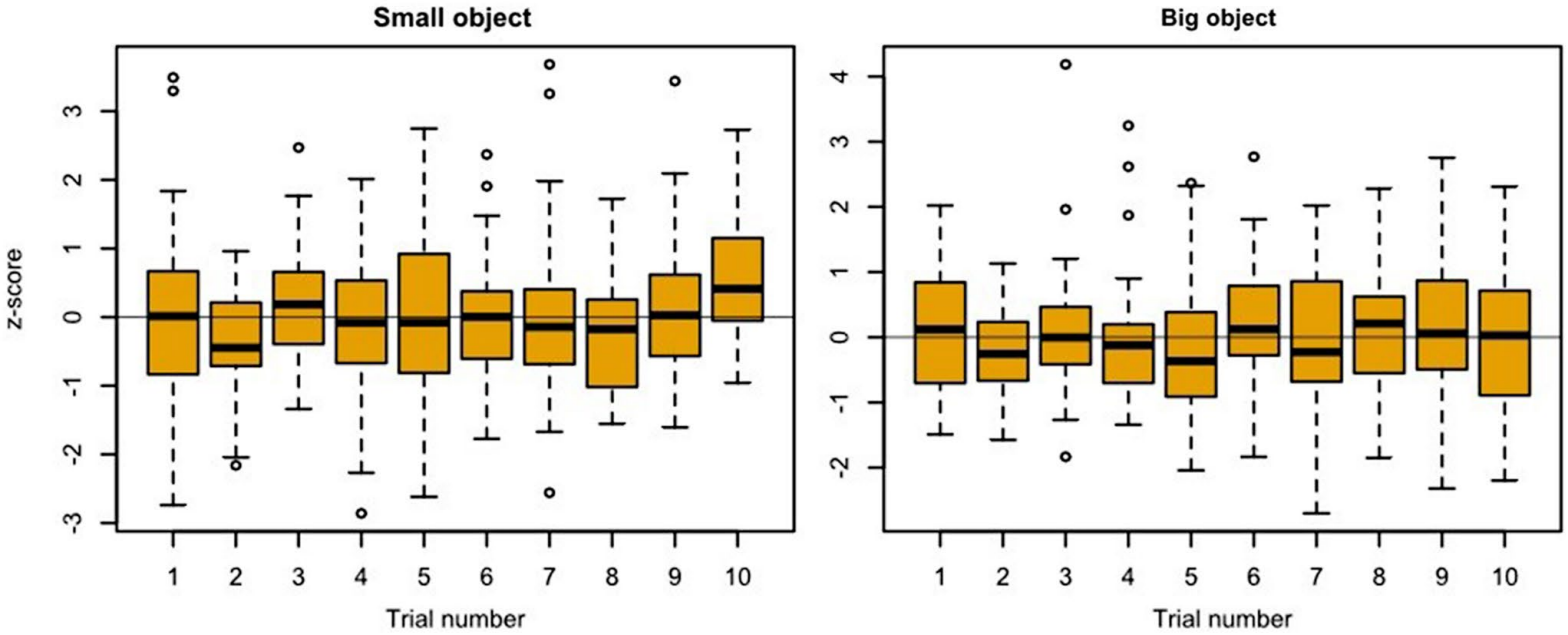
Z-score normalized receiver’s peak grip force rate data using medium-weight objects (700 g). The left column shows data using the small object. The right column shows data using the big object. The x-axis indicates the number of the measurement repetition of the object condition (medium and small/large), where 1 stands for the first presentation of the respective object condition and 10 for the tenth presentation. Values greater/smaller than 1.5 time of the interquartile range of the third/first quartile were marked by circles as outliers

Figure 5 shows the profiles of the grip force (Fig. 5, top), as well as the corresponding rates (Fig. 5, beneath) from one dyad for two trials using different object sizes (Fig. 5, left) and three trials using different object weights (Fig. 5, right). It can be seen that the giver’s peak grip force rate was lower for small objects than for large objects. In contrast, there was no clear demarcation in the giver’s peak grip force rate between the object weights. The giver’s peak grip force rate for light, medium and heavy weights were *M* = 104 (± 60 SD) N/s, *M* = 103 (± 57 SD) N/s, and *M* = 106 (± 62 SD) N/s. The normalized z-scores are shown in Fig. 5 (bottom). The ANOVA (Table 1) indicated that giver’s peak grip force rate was significantly higher for big objects than for small objects (F(1,39) = 8.33, *p* = .006, η2g = 0.14). No effect for object weight, (F(2,78) = 2.20, *p* = .118, η2g = 0.01) or interaction effect (size x weight; F(2,78) = 1.80, *p* = .172, η2g = 0.01) was found. The receiver’s peak grip force rates in Fig. 5 show the reverse effect between object properties and peak grip force rate compared to the giver’s grip force rates. While it is not possible to differentiate between object size on the basis of receiver’s grip force rates (Fig. 5, left), the peak grip force rate indicates a relation with object weight: The heavier the object, the higher the receiver’s peak grip force rate (Fig. 5, right). The receiver’s peak grip force rate for light, medium and heavy weights were *M* = 78 (± 42 SD) N/s, *M* = 85 (± 48 SD) N/s, and *M* = 89 (± 50 SD) N/s. The normalized z-scores are shown in Fig. 5 (bottom). The ANOVA (Table 1) revealed that the heavier the object is, the greater the receiver’s peak grip force rate (F(2,78) = 33.63, *p* < .001, η2g = 0.17), but no effect for object size (F(1,39) = 0.21, *p* = .647, η2g < 0.01) or interaction effect (size x weight; F(2,78) = 1.13, *p* = .327, η2g = 0.01). Figure 6 shows the receiver’s peak grip force rate for medium-weight objects over the course of the experiment. No change in grip force adaptation can be detected over the course of the experiment.

### Giver’s kinematics

**Fig. 7:**
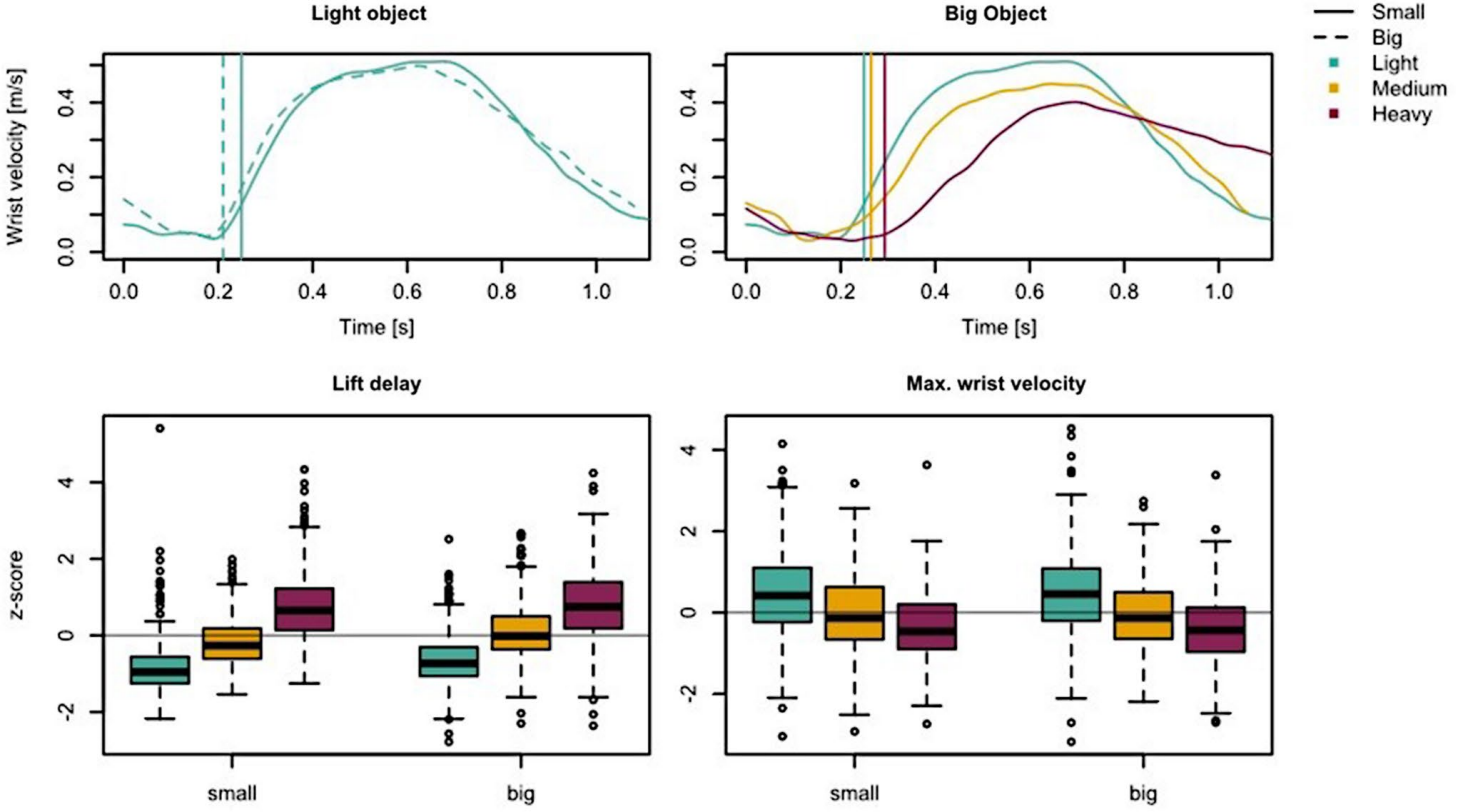
The plotted data are from one participant (giver) and synchronized to the time of initial contact between giver and object. Vertical lines indicate object lift-off. The left column shows two trials using the light object weight and the two different object sizes. The right column shows three trials using the big object and the three different object weights (400 g, 700 g, 1000 g). At the bottom are the Boxplots showing the z-score normalized lift delay and maximum lift velocity. Values greater/smaller than 1.5 time of the interquartile range of the third/first quartile were marked by circles as outliers

Figure 7 (top) shows the object’s lift-off and giver’s maximum lift velocity from one participant for two trials with different object sizes (Fig. 7, left) and three trials with different object weights (Fig. 7, right). The graphs are aligned to the initial contact between the giver and the object, a vertical line shows the onset of the object lift-off. The lift delay for light, medium and heavy weights were *M* = 249 (± 83 *SD*) ms, *M* = 311 (± 103 *SD*) ms, and *M* = 383 (± 135 *SD*) ms. The normalized z-scores are shown in Fig. 7 (bottom, left). The ANOVA (Table 1) revealed that the heavier the object, the longer the lift delay (F(2,78) = 387.77, *p* < .001, η2g = 0.76) and that the lift delay is longer for big objects than for small objects (F(1,39) = 5.18, *p* = .028, η2g = 0.07). No weight x size interaction effect was found (F(2,78) = 2.91, *p* = .061, η2g = 0.02). The maximum object transport velocity for light, medium and heavy weights were *M* = 0.47 (± 0.10 *SD*) m/s, *M* = 0.44 (± 0.09 *SD*) m/s, and *M* = 0.42 (± 0.08 *SD*) m/s. The normalized z-scores are shown in Fig. 7 (bottom, right). The ANOVA (Table 1) showed that the maximum velocity decreases with increasing object weight (F(2,78) = 120.71, *p* < .001, η2g = 0.46), but no effect for object size (F(1,39) = 0.04, *p* = .836, η2g < 0.01) or interaction effect (size x weight; F(2,78) = 0.17, *p* = .840, η2g < 0.01).

The post hoc analyses show significant effects between all weight conditions (*p* < .001). The effect sizes are listed in Table 2.

**Table 1:** Results of the analyses of variance for the parameters of interest

Predictor	df_Num_	df_Den_	Lift delay			Maximum lift velocity			Giver’s peak grip force rate			Receiver’s peak grip force rate		
			F	p	η^2^_g_	F	p	η^2^_g_	F	p	η^2^_g_	F	p	η^2^_g_
Weight	2	78	**387.77**	**<.001**	**.76**	**120.71**	**<.001**	**.46**	2.20	.118	.01	**33.63**	**<.001**	**.17**
Size	1	39	**5.18**	**.028**	**.07**	.04	.836	<.01	**8.33**	**.006**	**.14**	.21	.647	<.01
Weight × size	2	78	2.91	.061	.02	.17	.840	<.01	1.80	.172	.01	1.13	.327	.01

*Note. df*_*Num*_ indicates degrees of freedom numerator. *df*_*Den*_ indicates degrees of freedom denominator. η^2^_g_ indicates generalized eta-squared

**Table 2:** Results of the effect size analyses with Cohen’s d

Predictor	Object weight		
	Light-medium	Light-heavy	Medium-heavy
Lift delay	2.19	4.00	2.13
Maximum velocity	−1.29	−2.12	−0.99
Receiver’s peak grip force rate	.65	1.13	.43

## Discussion

This study investigated whether receivers in handover actions integrate information from observing the giver’s kinematics into their own action planning. The peak grip force rate was used as a measure of anticipatory grip force scaling (Hermsdörfer et al. 2011). The results showed that the giver determined a weight-independent peak grip force rate confirming that they had no prior contact knowledge about the object weight. In contrast, the receiver showed a peak grip force rate that was scaled to the object weight. Accordingly, the results support the hypothesis that the receiver generates information by observing giver’s kinematics and integrating them directly into their own action plan.

The results showed that the receiver, unlike the giver, is able to produce an initial grip force that is scaled to the object’s weight. This strongly implies that receivers are able to generate information about object weight by observing the giver’s kinematics as they lift the object. Previous studies have shown that visual cues and their integration with sensorimotor memory are used to make inferences about object weight, which in turn enables adequate grip force scaling (Buckingham et al. 2009; Fu and Santello 2012; Gordon et al. 1991c; Jenmalm and Johansson 1997; Schneider and Hermsdörfer 2016) or leads to reduced surprise effects (Meulenbroek et al. 2007). With the results of this study, we have shown that in addition to visual cues (such as size and material), observations of a co-actor may also be used to anticipate object weight. In the present study, the kinematics of the giver appear to be the main predictor of object weight as the receiver was able to produce an appropriate grip force despite the randomized presentation of various weights. This enables smooth and efficient transfer and less need for feedback processing.

Furthermore, it has previously been shown that receivers adapt their reaching kinematics to the giver kinematics during the transport phase (Mason and MacKenzie 2005). Our study extends these findings as we were able to show that different giver kinematics can also influence the peak grip force rate (not only the kinematics) of the receiver. It is important to note that the object weight in our study was changed in a randomized order, i.e., different object conditions were applied from trial to trial. Accordingly, it was not expected that receivers would learn to understand the giver’s kinematics over the course of the experiment. This is evident in our data as there is no improvement in the adaptation of the receiver’s peak grip force rates over the course of the experiment (see Fig. 6). Therefore, we argue that the receiver’s ability to estimate the weight is due to the mental representation of the giver’s action during the reach and grasp and object transport phases. These representations are continuously being integrated with the information monitored by observation up to the transfer phase, refining the estimates of the object’s weight. This interpretation is supported by assumptions of both, motor cognition (Jeannerod 2006) and joint action research (Sebanz et al. 2006), that during the action of another person, this action is represented by the observer, whereby the observer can generate information about intentions or (in the case of the present study) the object.

The result that the giver’s lift delay is primarily prolonged by an increasing object weight and, to a lesser extent, by object size is also in line with previous studies (Hermsdörfer et al. 2011; Johansson and Westling 1988; Weir et al. 1991). Similarly, the giver’s maximum lift velocity was shown to be influenced by weight variation (the heavier the object, the lower the maximum lift velocity). This is also consistent with previous studies (Johansson and Westling 1988; Kopnarski et al. 2023a).

Our results show that the giver’s peak grip force rate was influenced by the object size but not by the object weight, which is the most relevant property for the grip force scaling. The intention of the experimental design was that there should be no knowledge of the object weight prior to the start of a trial. If this is the case, no feedforward-controlled grip force scaling adapted to the object weight may be observed in the giver. Thus, the result that the object weight had no significant effect on the giver’s peak grip force rate is understood as proof of concept. The result that the object size had an effect on the giver’s peak grip force rate is in line with studies that have shown that object size influences the estimation of an object’s weight (Gordon et al. 1991a, c; Hermsdörfer et al. 2011; Johansson and Westling 1988) and with studies that have shown that if no accurate prediction about an object’s weight can be made, accurate initial grip force cannot be produced (Gordon et al. 1991a, b; Hermsdörfer et al. 2011; Johansson and Westling 1988).

Although the results of the present study clearly suggest that receivers observe givers in order to generate knowledge of the object, it is appropriate to note some potential limitations. It should be noted that, in this study, weight anticipation was not measured directly but indirectly via the peak grip force rate. Even though this study aimed to investigate whether it is possible for receivers to generate more accurate weight estimations by observing givers, the peak grip force rate of givers was also investigated. Hence, we know that the peak grip force rate of the giver is not adapted to the object weight but the peak grip force rate of the receiver is. From this, we can conclude that the receiver is provided with information about object weight through the giver’s kinematics, an opportunity that is not available to the giver prior to the initial contact. In order to investigate in more detail whether the difference between the peak grip force rates of givers and receivers is actually due to differences in object anticipation accuracy rather than the different initial situation from which the object is grasped (object standing on the table and must be lifted vs. object is grasped from an elevated position from the giver’s hand), we propose a possible operationalization for a future study. Another condition of object knowledge should be added in which object weight is presented blocked vs. randomized, similar to the studies undertaken by Weir et al. (1991). This would allow a direct comparison of how peak grip forces change in givers and receivers when knowledge of object weight is ensured. Another limitation is due to the giver’s uncertainty about the object’s weight. It is known that parameters such as lift delay and maximum lift velocity change with the accuracy of the anticipated object properties. More precisely, the better an actor anticipates the object weight, the smaller the differences in lift delay and maximum lift velocity will be between the different weights (Johansson and Flanagan 2009; Wing 1996). Therefore, the question arises whether receivers can also acquire information about the object weight if an object weight previously known to the giver is handed over. To investigate this question, we propose a study in which the giver is informed about the object weight prior to each trial but the receiver is not. This setup could be similar to the one used in the study by Endo et al. (2012) in which the giver was informed before each trial at which of three handover positions the object should be handed over. Furthermore, it should be noted that a purely horizontal handover situation was created in this study (see Fig. 3, start and end positions are on the same height). In everyday handover situations, it can be assumed that the object to be handed over is closer to the giver than to the receiver (i.e. also a vertical difference between start and end position). Possibly this synthetic handover situation influences the participants’ action strategy. For further studies, we recommend to change the start and end position of the object to be closer to the giver at the beginning and closer to the receiver at the end of handover.

The present study improves our understanding of the relationship between action observation and action planning. We confirm the findings from Meulenbroek et al. (2007) that estimates of object weight can be obtained by observing the kinematics of an actor in sequential replacement tasks and apply this to handover actions. Thus, we extend the knowledge of anticipatory grip force control by showing that weight estimates are not only made based on object properties (such as size and material), other environmental factors, such as observations of another actor’s kinematics during object manipulation, are also used.

## Data Availability

The data that support the findings of this study are available on request from the corresponding author.
