# Correction: Unveiling the invisible: receivers use object weight cues for grip force planning in handover actions

**DOI:** 10.1007/s00221-024-06835-6

**Published:** 2024-07-12

**Authors:** L. Kopnarski, J. Rudisch, D. F. Kutz, C. Voelcker-Rehage

**Affiliations:** Department of Neuromotor Behavior and Exercise Institute of Sport and Exercise Sciences, University of Münster, Münster, Germany

**Correction: Experimental Brain Research** 10.1007/s00221-024-06813-y

In Table 1 of this article, all less than signs (<) are inadvertently replaced by a character string &lt;. The incorrect Table 1 and the correct Table 1 is given below:

**Table 1:** Results of the analyses of variance for the parameters of interest

Predictor	df_Num_	df_Den_	Lift delay			Maximum lift velocity			Giver’s peak grip force rate			Receiver’s peak grip force rate		
			F	p	η^2^_g_	F	p	η^2^_g_	F	p	η^2^_g_	F	p	η^2^_g_
Weight	2	78	**387.77**	**&lt; 0.001**	**0.76**	**120.71**	**&lt; 0.001**	**0.46**	2.20	0.118	0.01	**33.63**	**&lt; 0.001**	**0.17**
Size	1	39	**5.18**	**0.028**	**0.07**	0.04	0.836	&lt; 0.01	**8.33**	**0.006**	**0.14**	0.21	0.647	&lt; 0.01
Weight × size	2	78	2.91	0.061	0.02	0.17	0.840	&lt; 0.01	1.80	0.172	0.01	1.13	0.327	0.01

*Note. df*_*Num*_ indicates degrees of freedom numerator. *df*_*Den*_ indicates degrees of freedom denominator. η^2^_g_ indicates generalized eta-squared

**Table 1:** Results of the analyses of variance for the parameters of interest

Predictor	*df*_*Num*_	*df*_*Den*_	Lift delay			Maximum lift velocity			Giver’s peak grip force rate			Receiver’s peak grip force rate		
			*F*	*p*	η^2^_g_	*F*	*p*	η^2^_g_	*F*	*p*	η^2^_g_	*F*	*p*	η^2^_g_
Weight	2	78	**387.77**	** < 0.001**	**0.76**	**120.71**	** < 0.001**	**0.46**	2.20	0.118	0.01	**33.63**	** < 0.001**	**0.17**
Size	1	39	**5.18**	**0.028**	**0.07**	0.04	0.836	< 0.01	**8.33**	**0.006**	**0.14**	0.21	0.647	< 0.01
Weight × Size	2	78	2.91	0.061	0.02	0.17	0.840	< 0.01	1.80	0.172	0.01	1.13	0.327	0.01

*Note. df*_*Num*_ indicates degrees of freedom numerator. *df*_*Den*_ indicates degrees of freedom denominator. η^2^_g_ indicates generalized eta-squared

